# Biomechanical Performance of Implant-Tooth-Supported Prostheses: A Numerical 3D Finite Element Analysis

**DOI:** 10.1016/j.identj.2025.103882

**Published:** 2025-09-10

**Authors:** Hatem S. Sadek, Noha M. Anany, Mohamed I. El-Anwar, Abdulaziz Alhotan, Al-Hassan Diab, Bassma R. Fayad, Islam M. Heiba, Islam G. Shahin, Christoph Bourauel, Tarek M. Elshazly

**Affiliations:** a Oral Technology, Dental School, University Hospital Bonn, Bonn, Germany; b Faculty of Dentistry, Ain Shams University, Cairo, Egypt; c Department of Mechanical Engineering, National Research Centre, Giza, Egypt; d Department of Dental Health, College of Applied Medical Sciences, King Saud University, Riyadh, Saudi Arabia; e Faculty of Dentistry, British University in Egypt, Cairo, Egypt; f Prothodontic Department, Private Clinic, Abu Dhabi, UAE

**Keywords:** Prosthodontics, Dental implant, Zirconia, Polymers, Finite element analysis, Stress analysis

## Abstract

**Objective:**

To assess the biomechanical behaviour of implant-tooth-supported (hybrid) prostheses, compared with an implant-implant-supported prosthesis, utilising two different prosthetic materials, employing finite element analysis (FEA).

**Method:**

Three digital models, derived from a CBCT-scanned edentulous mandible, were designed: two hybrid configurations (M1: distal implant-first premolar; M2: mesial implant-first molar) and an implant-implant-supported prosthesis (M3). A biomechanical modelling workflow was carried out (Mimics for segmentation, 3-Matic for bone/mucosal layer refinement, Exocad DentalCAD and SolidWorks for implants, abutment and prosthesis integration, and ANSYS for meshing and FEA). Assuming isotropic/elastic material properties, zirconia and polyetherketoneketone (PEKK) were selected as prosthetic materials. Models were subjected to a static vertical load (100 N). von Mises stress and total deformation were analysed to evaluate structural responses.

**Results:**

M3 demonstrated the most favourable biomechanical profile, exhibiting the lowest von Mises stress magnitudes alongside the highest overall deformation. In hybrid models (M1, M2), stress maxima were localised at the pontic–tooth connector regions and, in M1-PEKK, reached 261 MPa in the screw. Cortical bone and prepared teeth exhibited higher stress in distally anchored hybrid systems (M1) compared to mesially supported designs (M2), regardless of the prosthetic material. Comparative analysis revealed that zirconia-based prostheses generated higher stress concentrations in bone (≤35 MPa) compared to PEKK, whereas the PEKK prosthesis itself exhibited greater structural deformation (70 µm in M1-PEKK).

**Conclusion:**

Hybrid implant-tooth-supported prostheses can act as a reliable alternative to implant-implant ones and eliminate cantilever extensions; however, their use should be reserved for carefully selected cases to avoid biomechanical incompatibilities.

## Introduction

At present, implant therapy is recognised for its consistently high clinical success rates and its capacity to address numerous clinical challenges associated with edentulism, substantially enhancing patient quality of life.^1^, ^2^, ^3^ Nonetheless, long-term therapeutic efficacy and survival rate of dental implants are critically influenced by several biomechanical determinants, including bone tissue quality and volume in edentulous regions, the number and spatial distribution of placed implants,^4^ prosthetic superstructure design and material,^4^, ^5^, ^6^, ^7^ as well as patient-specific occlusion patterns and masticatory dynamics.^1^^,^^8^^,^^9^

Hybrid implant-tooth prostheses are selectively employed in cases of partial edentulism for indications such as free-end saddles, extensive alveolar defects, as well as anatomical or socioeconomic constraints.^10^^,^^11^ While concerns persist regarding biomechanical incompatibility between fastened osseointegrated implants and physiologically mobile natural teeth,^12^^,^^13^ longitudinal evidence supports their clinical feasibility, with no significant adverse biological impact.^14^, ^15^, ^16^ However, to optimise outcome, clinical protocols should exclude short implants, compromised bone quality and endodontically treated abutments while prioritising rigid connections and permanent cementation to minimise complications.^11^ Nevertheless, this approach holds risks, including osseointegration failure, abutment tooth damage and prosthetic complications,^11^ necessitating rigorous case selection and biomechanical optimisation.

Finite element analysis (FEA) is a prevalent technique for evaluating implant biomechanics in diverse clinical scenarios.^4^^,^^5^^,^^17^, ^18^, ^19^ By discretising complex geometries with heterogeneous materials into finite number of elements linked by nodes, FEA simulates structural responses with an appropriate mesh and mathematical model.^20^ It reliably predicts stress distributions on prosthetic components, implant systems and peri-implant bone,^4^^,^^21^ by this means it informs optimal design and material choices to improve clinical outcomes and implant longevity.^22^^,^^23^

Considering the aforementioned debate on the hybrid implant-tooth-supported prostheses, the current research employed FEA to assess the biomechanical performance of three different prosthetic models, two distinct designs of hybrid configurations compared with an implant-implant-supported prosthesis, using two different prosthetic materials under vertical loading. The first null hypothesis posited that there would be no differences in the mechanical behaviour of implant complex, prosthesis, cortical bone and PDL among the three models, while the second postulated that the choice of prosthetic material would not affect their biomechanical performance.

## Method

### Study design

The current study design was developed to evaluate three different prosthetic configurations at the mandibular posterior region. Models one (M1) and two (M2) featured fixed-fixed three-unit prostheses supported by one implant and one tooth abutment, while model three (M3) had a fixed-fixed three-unit prosthesis supported by two implants. In M1, the implant was placed at the first molar site, serving as the distal abutment, with the first premolar acting as the mesial tooth abutment. M2 reversed this configuration, positioning the implant at the first premolar site as the mesial abutment, while the first molar served as the distal tooth abutment. M3 incorporated two implants at the first premolar and first molar sites, supporting a central pontic at the second premolar site. The first premolar and molar were chosen as abutment sites due to their frequent use in posterior fixed partial dentures and their clinical relevance for replacing three-unit edentulous spans. For each design, either monolithic zirconia or PEKK was used as the prosthetic material, generating three models in each group: zirconia (M1-Z, M2-Z, M3-Z) and PEKK (M1-P, M2-P, M3-P).

### Creation of the geometric model

After obtaining approval from the Research Ethics Committee at the Faculty of Dentistry, Ain Shams University, Egypt (FDASU-ReclE121904), a cone beam computed tomography (CBCT) scan of an edentulous mandible was processed using Mimics 14 software (Materialise, Leuven, Belgium). The mandible was segmented to create a spongy bone core surrounded by a 2 mm-thick cortical shell. The segmented data was then exported as a standard tessellation language (STL) file to 3-Matic 7.01 software (Materialise, Leuven, Belgium) for refinement. In 3-Matic, the model was checked for geometric errors and smoothed. A 0.3 mm-thick periodontal ligament (PDL) as well as a 1 mm-thick mucosal layer were designed using the uniform offset operation.

Thereafter, CAD files of standardised prepared teeth (first lower premolar, first lower molar) (Digimation Corp., St Rose, Louisiana, USA), as well as tioLogic implant (Dentaurum, Ispringen, Germany)—consisting of a fixture (9.0 mm length, 3.7 mm width), an abutment and a fixing screw—were imported as STL files. The three models (M1, M2 and M3) were configured according to the study design and exported into Exocad DentalCAD software (Exocad, Darmstadt, Germany) to create the prosthetic suprastructure (Prosthesis). Finally, all models were exported in IGES format to SolidWorks V.2014 (Dassault Systèmes, Vélizy-Villacoublay, France) to correct any errors that may have occurred during the conversion from point cloud data to solid geometry.

### Mesh formation

The solid model components were exported as a STEP file for processing in FEA software (ANSYS Workbench 14.0; ANSYS, Canonsburg, PA, USA). Within the ANSYS environment, Boolean operations were applied to verify contact surface validity and finalise the geometrical models. The solid geometries were then discretised into finite elements using 3D brick solid elements (Element 187), guided by a mesh convergence test to ensure numerical accuracy.

**Fig. 1:**
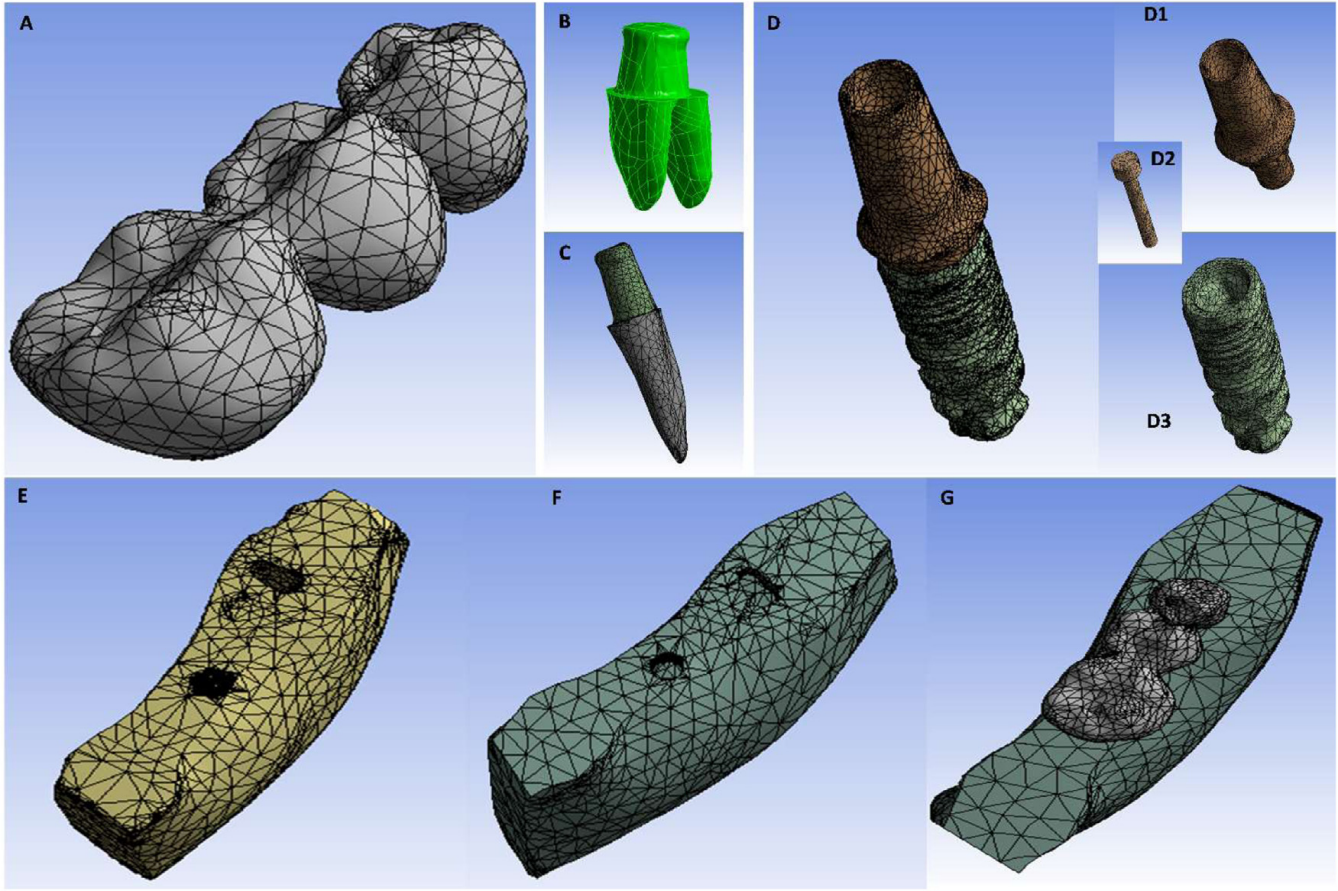
Components of the model: A, Three-units prosthesis. B, Molar tooth complex (prepared tooth and PDL). C, Premolar tooth complex (prepared tooth and PDL). D, Implants complex (Abutment [D1], Screw [D2] and Fixture [D3]). E, Compact bone. F, Spongy bone. G, Meshed model.

The mesh convergence test involved iteratively applying test loads to models with varying mesh densities, identifying the minimum element count required for reliable results, defined as cortical bone von Mises stress variations within ±5%. Convergence was achieved with a growth rate of 1.2 and element sizes ranging from 0.005 to 0.991 mm, effectively capturing complex anatomical details. The final meshes, generated in ANSYS, consisted of distinct element and node counts for each model (Table 1), with components’ images shown in Figure 1.

**Table 1:** Mesh density of the three models’ components.

	Model 1: T4+P5+I6		Model 2: I4+P5+T6		Model 3: I4+P5+I6	
Part/Material	Number of elements	Number of nodes	Number of elements	Number of nodes	Number of elements	Number of nodes
Three-units prosthesis	24,623	39,966	38,361	57,897	38,826	78,796
Abutment	23,130	44,655	23,572	45,661	16,518	28,968
Screw	20,465	35,947	20,490	35,972	22,252	38,286
Implant	3807	6750	3807	6750	7752	13,689
Prepared tooth	27,959	48,078	27,862	47,918	—	—
PDL	23,670	47,023	24,996	49,925	—	—
Soft tissue	8770	15,641	2215	4020	3590	6714
Cortical Bone	9146	16,214	8322	15,870	7256	13,464
Cancellous Bone	14,708	26,183	10,760	19,870	23,071	38,682

### Material properties, contact conditions and boundary conditions

All materials were considered isotropic, linear and elastic (Table 2). Glue contact was assigned to the interfaces between model components, assuming complete osseointegration at the bone-implant interface and cold welding between implant components.^24^ To ensure model stability, the mesial and distal boundaries of the bone volume were fixed in all three directions, restricting movement during load application. Model validation was performed by comparing the current models with those from similar studies before proceeding with numerical analysis and data extraction.^25^

**Table 2:** Properties of the experimental materials used in the finite element models.

Material	Modulus of elasticity in GPa	Poisson’s ratio	Reference
Prosthesis 1: Monolithic zirconia	200.00	0.26	Yousief et al^26^
Prosthesis 2: PEKK*	5.10	0.40	Alqurashi et al^27^
Implant	110.00	0.34	De Moor et al^28^
Prepared tooth	18.60	0.31	Yousief et al^26^
Periodontal Ligament (PDL)	0.10	0.30	Elshazly et al^29^
Soft tissue	10.00	0.40	Abaza et al^30^
Cortical bone	13.70	0.30	Al-Zordk et al^25^
Cancellous bone	1.37	0.30	Al-Zordk et al^25^

⁎ Polyetherketoneketone.

The mesial and distal surfaces of the bone were constrained in all three directions to prevent movement during load application, following the boundary conditions used in a recent study.^4^ Each model was subjected to a 100 N vertical load to the functional cusp of the pontic (second premolar). Linear static analyses were performed on an HP Z820 workstation (HP, Palo Alto, CA, USA) equipped with dual Intel Xeon E5-2660 2.2 GHz processors and 64 GB of RAM. von Mises stresses and maximum total deformation were calculated for various components across the models.

### Statistical analysis

FE simulations produce deterministic repeatable outcomes for defined input parameters, since they are constrained by governing equations and prescribed boundary conditions. Unlike experimental studies, which inherently involve variability from biological or experimental factors, FE solutions are uniquely determined for identical input parameters. Consequently, conventional statistical descriptors (eg, mean, standard deviation), which require data variability, are inherently irrelevant in FE modelling. Instead, sensitivity analysis (as detailed in the meshing protocol subsection of the method) provides a systematic framework to evaluate parameter influence and quantify uncertainty within FE systems.^20^

## Results

**Fig. 2:**
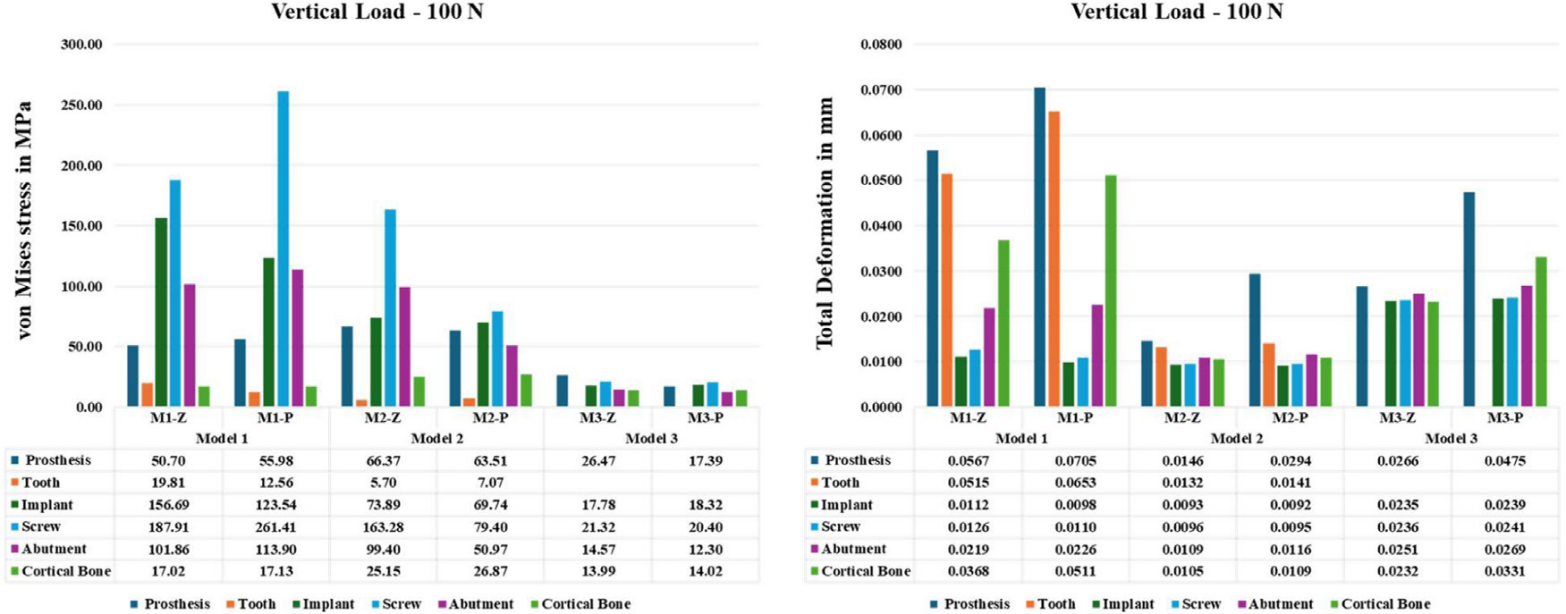
Comparison of equivalent von Mises stress (left) and total deformation (right) across different components under a vertical load of 100 N. Bar charts illustrate the mechanical response of various prosthetic and supporting structures—including prosthesis, tooth, implant, screw, abutment and cortical bone—across six model variations: M1-Z, M1-PEKK, M2-Z, M2-PEKK, M3-Z and M3-PEKK. Stress is presented in megapascals (MPa) and deformation in millimetres (mm), highlighting the influence of design (Model 1-3) and material (Zirconia vs PEKK) on biomechanical performance.

As shown in Figure 2, M3 models exhibited the most favourable mechanical performance, displaying the lowest von Mises stress values across all components.

**Fig. 3:**
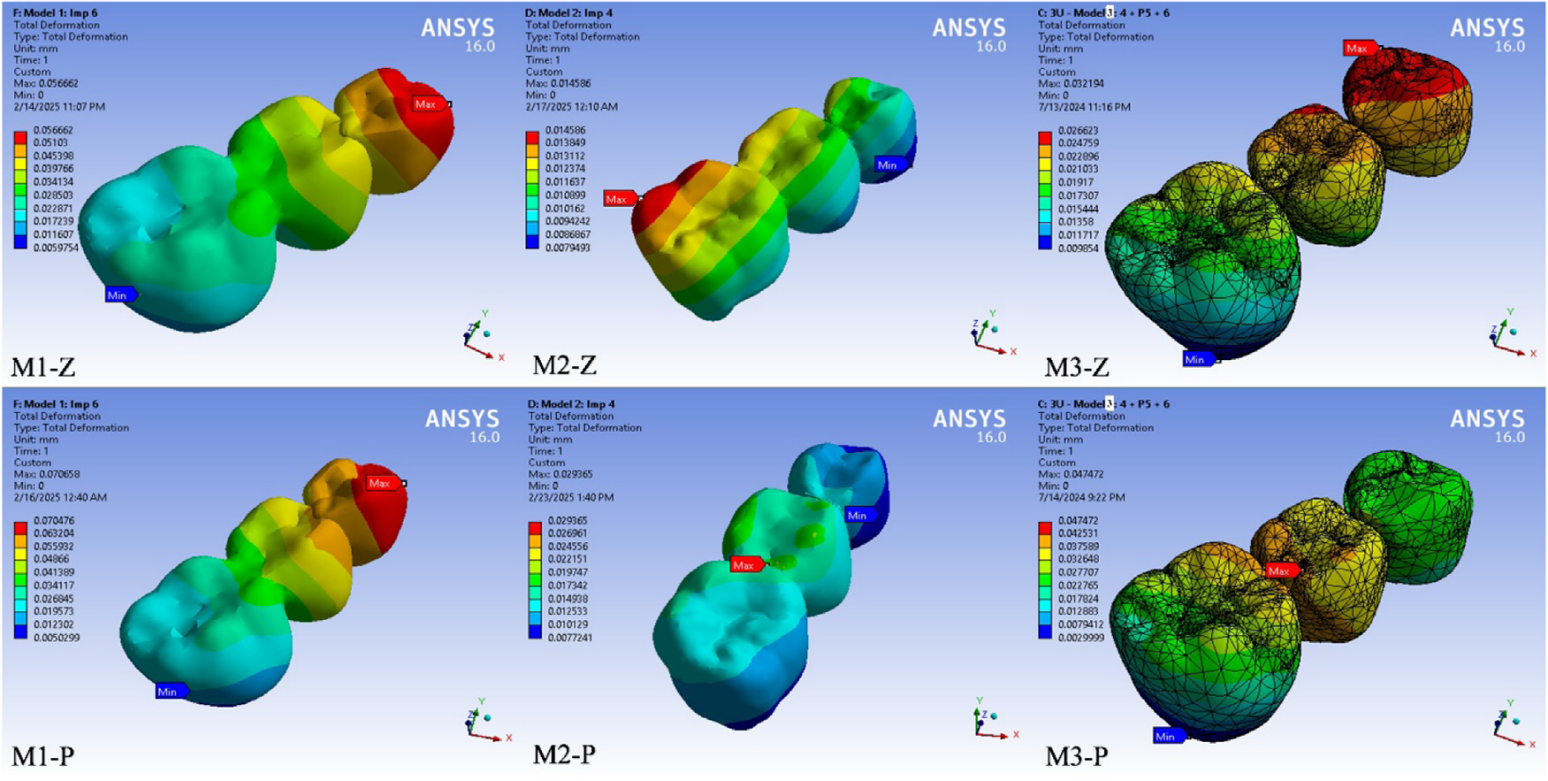
Color-coded finite element simulation results showing total deformation in the prosthetic body under vertical loading (100 N). Deformation distributions are visualised for each model–material combination: M1-Z, M1-PEKK, M2-Z, M2-PEKK, M3-Z and M3-PEKK. Warmer colours (red) indicate regions of maximum deformation, while cooler colours (blue) represent minimal deformation.

### Implant assembly

Within the implant assembly, the connecting screws consistently experienced the highest stress levels. Model M1-P reached a peak von Mises stress of 261 MPa in the screw. Maximum total deformation (7.05 µm) occurred at the superior surface of the abutment. In the implant body (Fixture), model M1-Z showed the highest stress concentration at 156.7 MPa.

### Prepared tooth and periodontal ligament (PDL)

For the prepared tooth, M1 designs yielded higher von Mises stresses than M2 designs; differences between zirconia and PEKK were minimal. In PDL, M1 consistently generated greater stress (0.24 MPa) and deformation (70 µm) than M2. The highest values were seen in M1-P, 0.24 MPa von Mises stress and 70 µm total deformation.

### Bone response

Cortical bone stresses remained moderate (≤35 MPa), with M2 models inducing nearly twice the stress (up to 26.9 MPa) compared to M3 (∼14 MPa), independent of material. Cancellous bone stresses were uniformly low (<13 MPa) across models. Soft tissue (mucosa) mechanical response was unaffected by either prosthetic design or material.

### Prosthetic body

von Mises stress hotspots in the prosthetic framework differed by design. M2 models yielded the highest prosthesis stresses (up to 66.4 MPa), while M3 models showed the lowest (down to 17.4 MPa). In M1 and M2, highest stresses localised at the connector between the central pontic and the tooth abutment. While in M3, Peak stresses centred on the pontic itself. These patterns were independent of zirconia versus PEKK. Overall, M2 produced the highest prosthetic stresses and M3 the lowest, with no significant material effects within each model.

### Total deformation

**Fig. 4:**
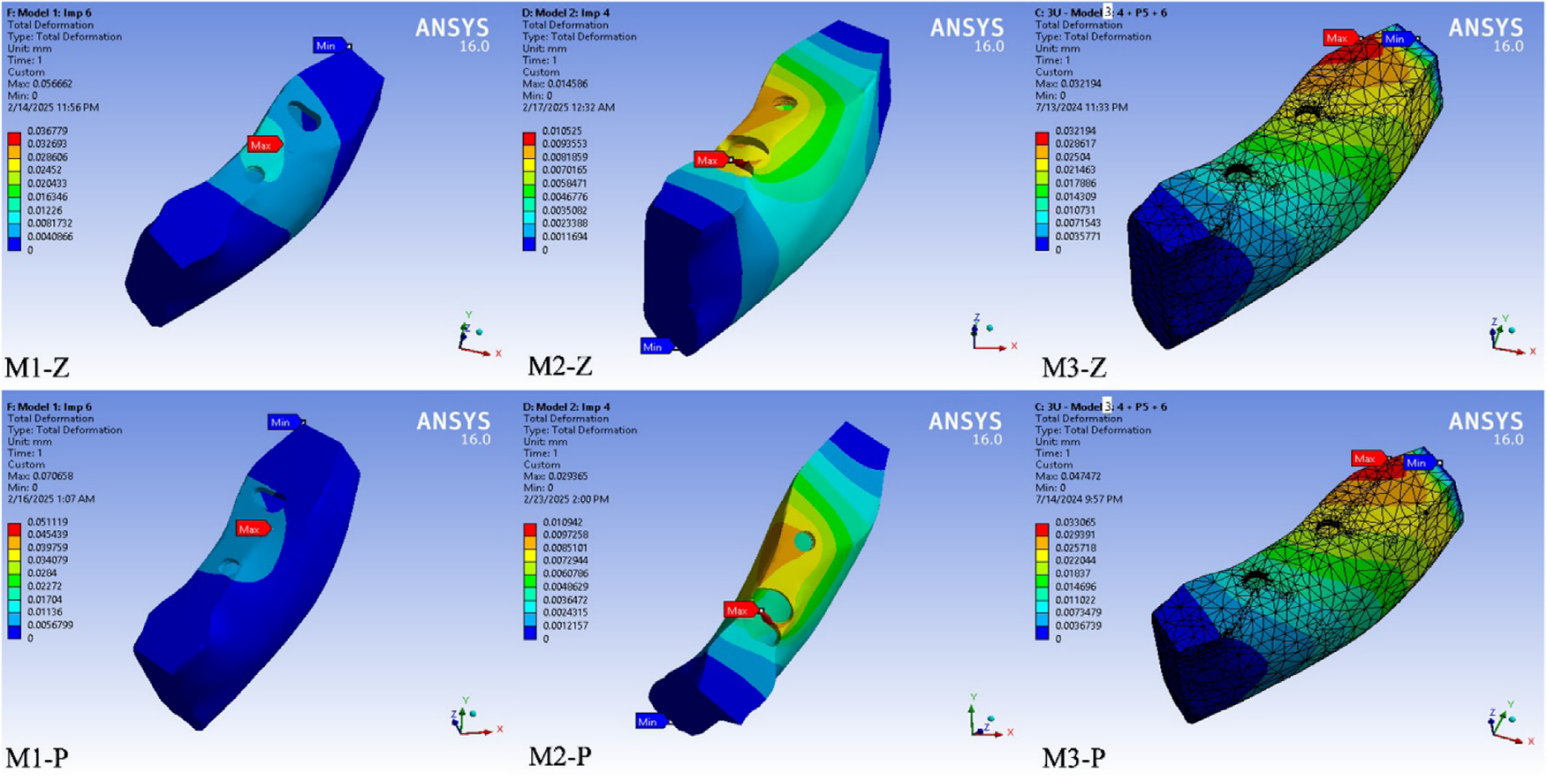
Color-coded finite element simulation results showing total deformation in the cortical bone under vertical loading (100 N). Deformation distributions are visualised for each model–material combination: M1-Z, M1-PEKK, M2-Z, M2-PEKK, M3-Z and M3-PEKK. Warmer colours (red) indicate regions of maximum deformation, while cooler colours (blue) represent minimal deformation.

As shown in Fig. 2, Fig. 3, Fig. 4, in prosthesis and cortical bone, M1 models deformed significantly more than M2 and M3. In implant complex, M3 models exhibited the largest deformation compared to M1 and M2. Generally, PEKK prostheses deformed more than zirconia counterparts in all cases. Maximum deformation values recorded were 70 µm for M1-P, 29 µm for M2-P and 47 µm for M3-P. The prepared tooth likewise deformed more in M1 designs than in M2, especially with PEKK.

## Discussion

Current literature presents divergent perspectives regarding the biomechanical behaviour and clinical reliability of hybrid tooth–implant-supported prostheses. Multiple studies^14^^,^^16^^,^^31^^,^^32^ have reported favourable functional outcomes, high patient satisfaction and comparable survival rates of implants and prostheses in hybrid systems relative to fully implant-supported designs. On the contrary, critics^12^^,^^33^, ^34^, ^35^ emphasise biomechanical incompatibility arising from the differential mobility of osseointegrated implants versus periodontally suspended teeth. Specifically, it has been demonstrated that a natural tooth with an intact periodontal ligament can exhibit displacements of up to 200 μm under a 0.1 N load, whereas a dental implant undergoes less than 10 μm of displacement under similar conditions, primarily due to bone flexure.^32^ This advocates restriction of connecting teeth to implant to limited cases involving anatomical limitations, implant failure, or socioeconomic constraints.

The present study demonstrated that Model 3 (implant-implant-supported prosthesis) exhibited superior mechanical performance, characterised by the lowest von Mises stress values compared to Models 1 and 2 (tooth–implant-supported designs). These findings align with those of Renouard et al,^36^ who highlighted that discrepancies in mobility between implants and teeth could result in the natural tooth being displaced within its socket, effectively converting the implant into a cantilever fulcrum. Meaning that under off-axis loading, the implant functions as a cantilevered support, resulting in increased stress concentrations, particularly at the implant–bone interface. This scenario potentially increases the mechanical load on the implant, predisposing it to technical complications and peri-implant bone overload. Conversely, Fardal et al^15^ found that the combination of teeth and implants did not compromise the performance of cross-arch stabilising prostheses in periodontally compromised patients and was associated with low complication rates and minimal loss of abutment teeth. Similarly, a meta-analysis by Huang et al^37^ reported no statistically significant differences between tooth–implant-supported and fully implant-supported prostheses in terms of prosthesis or implant failure rates, technical complications, or marginal bone loss.

In the current simulation, Models 1 and 2 showed that the natural tooth component could support the prosthesis with lower stress concentrations compared to the titanium implant complex. This can be attributed to the biomechanical cushioning effect of PDL, which functions as a shock absorber. This observation is consistent with findings from Li et al^38^ and Yousief et al^26^ who noted that natural teeth generate lower stress levels than implants under functional loading. Li et al^38^ also reported that stress distribution around natural teeth is more diffuse, extending from the apical to the cervical regions, whereas implants substantially alter stress transmission within the alveolar bone. Menicucci et al^39^ further observed that prolonged static loads may pose a greater risk to peri-implant bone than to alveolar bone due to the absence of PDL in implants, underscoring the PDL’s role in effective stress distribution.

Additionally, Davies et al^12^ posited that the absence of PDL around implants compromises their ability to accommodate non-axial forces. To address this mismatch, several researchers^40^^,^^41^ have introduced non-rigid connectors in tooth–implant prostheses to allow for minor tooth movement within the alveolar socket, promoting more uniform stress distribution and compensating for the limited implant mobility. In other studies,^42^^,^^43^ it has been suggested that natural teeth in hybrid prostheses can share occlusal loading with the implant, reducing the implant’s load-bearing burden. This load-sharing mechanism is believed to stem from the inherent bending flexibility of the implant–abutment joint, which mimics the axial compliance of the periodontal ligament.^43^

With respect to prosthetic material, implants supporting rigid zirconia bridges were subject to higher von Mises stresses compared to those supporting the more compliant PEKK bridges, in agreement with the findings of Yousief et al.^26^ On contrary, Kümbüloğlu et al^44^ reported that different superstructure materials had no significant influence on stress distribution within the implant system. Despite the observed variations, the maximum stress levels in the current study (156 MPa in model M1-Z) remained well within physiological limits, and below half the yield strength of titanium (520 MPa)^45^ indicating acceptable safety margins. In contrast, the screw stress in M1-PEKK reached 261 MPa, approaching the plastic deformation threshold of commercially pure titanium; thus, clinicians may consider using reinforced titanium alloys such as Ti-6Al-4V or adopting shorter-span prosthetic designs to mitigate the risk of mechanical failure.

This study has several limitations. First, PEKK and zirconia were modelled as isotropic, linearly elastic materials. However, their clinical behaviour may differ significantly: PEKK exhibits viscoelastic properties under cyclic loading, while zirconia is prone to brittle fracture under tensile stress. Although such simplifications are common and necessary in standard finite element analysis,^46^ future studies should incorporate non-linear and time-dependent material properties to enhance physiological accuracy and predictive reliability. Second, a standardised vertical static load of 100 N was applied; however, actual masticatory forces typically involve dynamic and oblique forces. Therefore, future research should incorporate oblique and cyclic loading conditions to more accurately assess the mechanical behaviour and long-term resilience of hybrid prostheses under clinically relevant scenarios. Third, the consistent stress concentration at the pontic–tooth connector, particularly in M1 and M2, underscores the biomechanical sensitivity of this region. While a uniform connector design was used across all models for standardisation, future studies could investigate variations in connector geometry—such as cross-sectional area, height and radius—to identify designs that better mitigate stress transmission.

## Conclusions

Within the limitations of this study, implant–implant-supported prostheses (Model 3) demonstrated the most favourable biomechanical performance, with the lowest von Mises stress values and minimal deformation across all components, suggesting they should be prioritised when clinically feasible. Tooth–implant-supported prostheses may offer a practical alternative by reducing implant count and avoiding cantilever extensions, but their use should be restricted to well-selected cases due to the risk of biomechanical mismatch. Natural teeth, through the cushioning effect of the periodontal ligament (PDL), exhibited lower stress concentrations compared to titanium implants and played a critical role in modulating occlusal force transmission to alveolar bone. Zirconia frameworks, though rigid, were associated with greater stress transfer to implant components, whereas PEKK exhibited higher deformation but lower stress concentrations. Cancellous bone and oral mucosa showed minimal sensitivity to changes in prosthetic material, emphasising that prosthetic design and support configuration play a more significant role than material choice in determining biomechanical outcomes.

### Clinical significance

The findings of this study guide prosthodontists and implantologists in selecting optimal designs and materials for hybrid implant–tooth-supported prostheses. These restorations may be suitable in cases with anatomical limitations, implant failure, or financial constraints, but should be avoided in patients with high parafunctional activity, severe periodontal issues, or tooth mobility. Rigid connections and precise occlusal design are crucial to minimise stress at the implant–tooth interface. The results indicate that hybrid prostheses can be a viable alternative to implant-only solutions, particularly when cantilevers are contraindicated. Mesial implant placement proved more favourable than distal placement in reducing stress. PEKK, with its greater flexibility, may help protect peri-implant bone in high-risk patients, while zirconia provides superior strength and aesthetics. Overall, the study supports a personalised approach to treatment planning to enhance long-term success and minimise biomechanical complications.

## Author contributions

**HS:** Methodology, Investigation, Data curation, Validation, Writing – original draft, Writing – review & editing. **NA:** Methodology, Investigation, Data curation, Validation, Writing – original draft, Writing – review & editing. **MIE:** Methodology, Investigation, Data curation, Validation, Writing – review & editing. **AA:** Methodology, Data curation, Validation, Writing – review & editing. **AHD:** Methodology, Data curation, Validation, Writing – review & editing. **BF:** Methodology, Data curation, Validation, Writing – review & editing. **IH:** Methodology, Data curation, Validation, Writing – review & editing. **IS:** Methodology, Data curation, Validation, Writing – review & editing. **CB:** Methodology, Data curation, Validation, Resources, Writing – review & editing. **TME:** Methodology, Investigation, Data curation, Validation, Writing – original draft, Writing – review & editing. All authors have read and agreed to the published version of the manuscript.

## Ethics statement

This study was approved by the Committee of Ethics in Research at the Faculty of Dentistry, Ain Shams University, Egypt (FDASU-ReclE121904).

## Conflict of interest

The authors declare that they have no known competing financial interests or personal relationships that could have appeared to influence the work reported in this paper.
